# Can Advances in Artificial Intelligence Strengthen the Role of Intraoperative Radiotherapy in the Treatment of Cancer?

**DOI:** 10.3390/cancers17193124

**Published:** 2025-09-25

**Authors:** Marco Krengli, Marta Małgorzata Kruszyna-Mochalska, Francesco Pasqualetti, Julian Malicki

**Affiliations:** 1 Department of Imaging and Radiotherapy, Veneto Institute of Oncology IOV IRCCS, 35128 Padua, Italy; 2 Department of Surgery, Oncology and Gastroenterology, University of Padua, 35122 Padova, Italy; 3 Electroradiology Department, Poznan University of Medical Sciences, 61701 Poznan, Poland; 4 Greater Poland Cancer Centre, 61866 Poznan, Poland; 5 Department of Biomedical Physics, Adam Mickiewicz University, 61712 Poznan, Poland

**Keywords:** radiotherapy, intraoperative radiotherapy, intraoperative electron radiotherapy, IORT, IOERT, artificial intelligence

## Abstract

Intraoperative radiotherapy (IORT) is a radiation technique that allows for the delivery of a high radiation dose to the target while preserving the surrounding structures, which can be displaced during the surgical procedure. An important limitation of this technique is the lack of real-time image guidance, which is one of the main achievements of modern radiation therapy because it allows for treatment optimization. IORT can be delivered by low-energy X-rays or by accelerated electrons. The present review describes the most relevant clinical applications for IORT and discusses the potential advantages of using artificial intelligence (AI) to overcome some of the current limitations of IORT. In recent decades, IORT has proven to be an effective treatment in several cancer types. In breast cancer, IORT can be used to deliver a single dose of radiation (partial breast irradiation) or as a boost in high-risk patients. In locally advanced rectal cancer, a single dose to the tumor bed can improve local control and prevent pelvic relapse in primary and recurrent tumors. In sarcomas, IORT enables the delivery of high doses, achieving good functional outcomes with low toxicity in tumors located in the retroperitoneum and extremities. In pancreatic cancer, IORT shows promising results in borderline resectable and unresectable cases. Ongoing technological advances are addressing current challenges in imaging and radiation planning, paving the way for personalized, image-guided IORT. Recent innovations such as CT- and MRI-equipped hybrid operating theaters allow for real-time imaging, which could be used for AI-assisted segmentation and planning. Moreover, the implementation of AI in terms of machine learning, deep learning, and radiomics can improve the interpretation of imaging, predict treatment outcomes, and optimize workflow efficiency.

## Simple Summary

Intraoperative radiotherapy (IORT) is a technique used to deliver a concentrated dose of radiation during surgery, allowing for the precise targeting of cancerous tissues while minimizing exposure to healthy structures. This review explores the clinical applications of IORT and the limitations related to the lack of image-guided treatment planning. We review recent developments in artificial intelligence (AI)—including machine learning, deep learning, and radiomics—that may further enhance IORT by improving image analysis, predicting treatment outcomes, and optimizing workflow efficiency. Integrating AI in IORT may lead to more personalized and effective cancer care, reduce treatment variability, and improve clinical decision-making.

## 1. Introduction

Intraoperative irradiation is a procedure by which radiation is delivered to a target during surgery to improve local tumor control while limiting irradiation of the skin and superficial tissues. This procedure was first performed more than 120 years ago in a woman with endometrial cancer. Since that time, a series of major technological advances have led to the increasing clinical use of intraoperative radiotherapy (IORT) around the world. From the 1930s to the 1950s, low-energy X-rays were used to treat abdominal, thoracic, and head and neck cancers. In the 1960s, Cobalt gamma rays and betatron electrons were used to deliver a single, high dose of radiation, an approach that was first performed at Kyoto University in Japan by Abe et al. [1]. In the 1970s, two hospitals in the United States (Howard University Hospital and Massachusetts General Hospital) used conventional linear accelerators for IORT. In the 1990s, dedicated mobile electron linear accelerators and miniaturized low-energy X-ray machines first became commercially available and introduced into routine clinical practice at several centers around the world [1,2]. Later, a new intraoperative modality based on megavoltage electrons was developed. That technique eventually became known as intraoperative electron radiotherapy (IOERT). The main difference between X-rays IORT and IOERT is the dose distribution at the target. IORT uses spherical applicators to deliver low-energy X-rays (20–50 KeV). By contrast, IOERT delivers accelerated electrons, with higher energies (ranging from 3 to 15 MeV). These two techniques also differ in terms of the penetration depth, which is only 0.5–1.0 cm from the surface of the IORT applicator but approximately up to 4–5 cm for IOERT, depending on the electron energy and reference isodose. The main characteristics and differences between these two intraoperative techniques are shown in Table 1. Although both kilovoltage IORT and IOERT have been shown to yield excellent treatment outcomes, there is an intense, ongoing debate in the field of radiation oncology among experts and opinion leaders with regard to which of these two techniques is superior [3]. A challenge related to intraoperative irradiation is that it requires a multidisciplinary staff with specific professional skills related to this specific treatment modality and the need for expensive equipment and professional expertise [4].

In an effort to promote the clinical use of this innovative treatment modality, the International Society of Intraoperative Radiation Therapy (ISIORT) was founded in 1998 to foster sharing of clinical experience among treatment centers and. The European section of the ISIORT (ISIORT-Europe), which was created in 2006, established a database registry in that same year [5,6]. The whole purpose was to collect and record technical and clinical data related to IORT at the affiliated centers. Analyses of the data from the ISIORT registry provide a clear picture of the patterns of care in IORT among the participating centers (n = 46, mostly in Europe, but also in Asia and Central America), including the IORT technique, the main tumor types, and patient selection criteria. A presentation at the 2021 meeting of the European Society for Radiotherapy and Oncology (ESTRO) reported results from a study of data from approximately 15,000 patients that showed that breast cancer accounted for the vast majority (81%) of IORT treatments, followed by rectal cancer, sarcoma, prostate, and pancreatic cancers [7]. Over time, numerous clinical studies of retrospective cohorts have been performed, with pooled analyses from experienced centers. In some tumor types, randomized clinical trials have been conducted. The ESTRO-ACROP has published recommendations for the use of IORT in selected tumor types. As a result, IORT is widely recognized for its capacity to achieve dose intensification to maximize the local control (in combination with surgery) while limiting the potential side effects associated with external beam radiation, which can harm the surrounding structures.

According to some experts, one disadvantage of both kilovoltage X-ray IORT and megavoltage IOERT is that dose distributions must be determined in real-time (i.e., during treatment), which may lead to inaccuracies. In all other modern radiotherapy treatment modalities, by contrast, the dose distribution is precisely determined prior to treatment. The dose distribution is essential for patient positioning and to ensure accurate clinical decision-making. In IORT/IOERT, it is not possible to determine the dose distribution without 3D images obtained immediately after surgery but before the initiation of IORT. Moreover, in the operating room, there is only a short window of time to perform dose optimization following surgery. However, computed tomography (CT) scanners are now available in the operating rooms at a growing number of centers, thus overcoming this important obstacle to dose distribution optimization. In recent years, the application of artificial intelligence (AI) in oncology has revolutionized the diagnostic accuracy and efficiency of imaging. Advanced algorithms, particularly machine and deep learning models (a type of AI), are now able to analyze complex imaging data, identifying subtle patterns present in tumors that the human eye is unable to detect. In oncology, these algorithms may help to improve patient selection for specific treatment types, including IORT [8]. In the near future, advances in computational capacity applied to radiotherapy are expected to allow medical physicists to quickly and accurately calculate dose distributions, even under the demanding conditions of the operating room.

Given this background, the purpose of this article is to comprehensively review intraoperative radiotherapy in the context of recent technological advances. More specifically, the aims are to (1) describe the most important clinical data for IORT, and (2) explain how advances in computational capacity and large imaging datasets will permit faster and more reliable processing for segmentation (contouring), dose distribution calculations, and evaluation.

## 2. Main Clinical Achievements of IORT

The main rationale for IORT is that it only requires a single, high dose of radiation while simultaneously limiting radiation exposure to the surrounding structures, which can be displaced during surgery to avoid unnecessary irradiation. Moreover, the energy of electron beams can be easily set up to minimize the dose beyond the target. In addition to this advantage in spatial selectivity, in recent years another highly intriguing biological rationale has emerged: several studies have shown a positive influence on the local microenvironment, which can improve local immunity to potentially reduce the risk of local recurrence [9,10,11,12]. In fact, recent studies support further research into the role of single high-dose radiation with immunotherapy to increase long-term disease control and to promote abscopal effects on distant tumor sites [13,14].

IORT has been used to treat a wide range of cancer types, with two main but different purposes. First, to achieve dose intensification by combining IORT with EBRT to improve local control in locally advanced or recurrent tumors (e.g., rectal cancer, sarcoma, pancreatic cancer, among others). Second, as an alternative to other radiation modalities (e.g., EBRT) with a proven capacity to successfully treat certain tumor types. In this case, the entire radiation treatment can be performed with IORT during surgery to reduce the total treatment time. The best example of this approach is seen in early breast cancer, whose efficacy and safety has been confirmed at specialist IORT centers through non-inferiority trials of single shot irradiation.

To better characterize the main indications for IORT we reviewed the available literature and reported a descriptive review including prospective clinical trials, major case series, meta-analysis, and systematic reviews. The findings of this literature review are summarized in Table 2 and described in more detail in the paragraphs below.

### 2.1. IORT in Breast Cancer

Numerous centers now use IORT as a routine treatment for breast cancer. In some centers, IORT is administered as the single radiation modality, which has the advantage of reducing overall local treatment time, thus improving quality of life, while also reducing waiting lists. In other centers, IORT is administered as a boost in combination with EBRT to improve local control (LC) in high-risk patients (Table 2).

The feasibility and the efficacy of single-shot IORT, a type of partial breast irradiation (PBI), has been studied in well-selected patients [29,30]. The ESTRO and the American Society for Radiation Oncology (ASTRO) have separately developed criteria to guide treatment indications for PBI and accelerated PBI (APBI) [31,32,33]. Numerous studies have described the use of IORT—whether delivered through electrons or low-energy X-rays—in the treatment of breast cancer, either as a single shot modality or combined with EBRT.

The ELIOT trial compared IOERT (21 Gy) to whole breast irradiation (WBI) in women with early breast cancer. Although in-breast tumor recurrence (IBTR) was higher in the IOERT group compared to the WBI group (15-year IBTR: 12.6% vs. 2.4%, *p* < 0·0001), there were no significant differences between the groups in overall survival (OS), nor were there any differences in IBTR in the low-risk patients. Based on those findings, the authors concluded that IOERT could be offered to selected patients at low risk of IBTR [16,34]. A few studies have also reported encouraging results with the administration of a single fraction to the surgical bed after local tumor recurrences [35]. A metanalysis compared PBI to non-IORT techniques, IORT, and WBI in early-stage disease, finding no differences in IBTR between PBI and WBI while IORT was inferior to WBI [36].

Fastner et al. performed a pooled analysis of data from the ISIORT centers, which showed that IOERT administered as a boost for dose intensification reduced local recurrence. In that study, a 10 Gy IOERT boost delivered prior to WBI yielded a local tumor control rate of 99.2% at a median of 72.4 months of follow-up, which was superior to the LC rates obtained in similar studies without IORT [15]. More recently, the ESTRO-ACROP (Advisory Committee for Radiation Oncology Practice) conducted a review of published data on IOERT, concluding that IOERT should be considered as a boost technique in higher risk patients. That review also showed that single shot IOERT is a feasible APBI technique for patients with low-risk disease, despite mild acute and late toxicity [35]. A metanalysis showed that IORT boost + WBI was more effective than conventional radiotherapy after breast-conserving surgery, with a similar cosmetic and safety profile to that of conventional radiotherapy [37]. The Targit-A trial found no differences between IORT (low-energy X-rays) and WBI in terms of local recurrence-free survival, survival without mastectomy, survival without distant relapse, or breast cancer-related mortality [17]. Moreover, 5-year LC rates were 94.15% for TARGIT-IORT vs. 94.19% for EBRT, with breast conservation rates of 92.32% and 91.64%, respectively. A subsequent metanalysis compared LR rates in patients treated with and without neoadjuvant IORT (low-energy X-rays), finding no differences between the groups [38].

In short, the available clinical data clearly show that IORT—whether delivered with electrons or low-energy X-rays—delivered as PBI, is a safe alternative to EBRT in patients with low-risk breast cancer and as a boost in patients at higher risk of local recurrence.

### 2.2. IORT in Rectal Cancer

In rectal cancer, studies have shown that a single, high dose of IOERT to the tumor bed can improve local control and prevent pelvic relapse in locally advanced primary tumors. IOERT can also be used to treat recurrent tumors (Table 2). This is important given that up to 50% of these patients develop metastatic disease, which has a poor prognosis. Several studies have shown that IOERT improves local control in locally advanced primary tumors and recurrent tumors [18,39,40]. A pooled analysis of 605 patients with locally advanced rectal cancer (LARC) who underwent multimodal treatment—including IOERT—found low local recurrence rates (12%) at 5 years, even though these patients were considered high risk [19]. In the same study, distant metastasis and OS rates were 29.2% and 67.1%, respectively.

To date, only two randomized trials have compared IOERT to other treatment modalities in relatively low risk patients (i.e., those who are unlikely to benefit from IOERT) [21,41]. Dubois et al. conducted a small trial to compare conventional preoperative EBRT to EBRT plus IOERT; however, there were no significant differences in treatment outcomes between the two groups [21]. In 2020, Masaki et al. reported the results of a randomized trial involving patients with LARC. That trial compared oncologic outcomes in patients who underwent surgery with IORT and bilateral pelvic autonomic nerve preservation (treatment arm) versus no IORT and limited nerve preservation (controls). The trial was closed early because the interim analysis showed that distant metastasis-free survival rates were significantly lower in the IORT arm, without significant between-group differences in OS or LC [41].

In the last decade, several systematic reviews and meta-analyses have been performed to clarify the role of IOERT in LARC [42]. In 2020, the ESTRO-ACROP published recommendations for the use of IORT in locally recurrent rectal cancer based on a comprehensive review of the literature, which included 2843 patients from 21 different studies, with reported LC rates ranging from 35% to 72% [43]. Based on the findings of that review, IOERT was recommended in patients with potentially resectable stage T3–T4 disease after preoperative chemotherapy + EBRT in case of gross residual disease or microscopically positive or close (< 2–5 mm) soft tissue margins. The total recommended doses were as follows: R0: 10–12.5 Gy; R1: 12.5–15 Gy; R2: 15–20 Gy. The NCCN guidelines (v. 3.2024) also recommend IORT as a boost in patients with very close or positive margins, particularly in stage T4 or recurrent cancers.

### 2.3. IORT in Sarcomas

IOERT can be considered part of the multimodal treatment of sarcoma due to its ability to deliver a high dose of radiation to the target volume [30]. The potential advantage of IOERT in this setting is that only a limited volume of healthy tissue is exposed to high dose radiation, which is crucial given the negative correlation between irradiation of healthy tissue and late toxicity (e.g., edema, fibrosis and impaired joint function), which can lead to poor functional outcomes [44,45]. An intraoperative boost can be delivered under visual control precisely to the high-risk region, without the need for additional margins to compensate for daily setup uncertainties. Moreover, key structures—including the peripheral nerves, great vessels, and skin—can all be excluded from the target volume, further reducing the risk of late toxicity [46]. Several reviews have shown that IOERT can achieve high control rates in selected cases of soft tissue sarcoma of the trunk and extremities [22,47,48,49,50]. Table 2 summarizes the findings of relevant studies and clinical trials.

Studies in patients with retroperitoneal sarcomas have consistently shown that preoperative EBRT + IOERT achieves 5-year LC rates ranging from 51% to 89%, which compare favorably to surgery alone and surgery + EBRT [51]. A randomized trial by the US National Cancer Institute compared surgery + IORT to the conventional approach consisting of surgery in combination or not with postoperative EBRT showing significantly improved local control with similar late gastrointestinal toxicity [22]. In general, preoperative radiotherapy should be preferred to postoperative radiotherapy due to better LC rates and less toxicity [52]. A recent single-arm, phase I-II dose escalation study was performed in patients with retroperitoneal sarcoma, many of whom had grade (G3) lesions and leiomyosarcoma. Treatment included preoperative IMRT, surgery, and IOERT. Five-year LC and OS rates were 59.6% and 59.5%, respectively, in the whole cohort, but LC rate was higher (64.8%) in the group of patients treated with IORT [25]. The results of the EORTC STRASS trial showed that preoperative irradiation can improve abdominal recurrence-free survival in patients with primary, well-differentiated liposarcoma and G1-2 dedifferentiated liposarcomas, although only some patients with sarcoma benefitted from this treatment [53].

The main aim of combined radiotherapy (IOERT + EBRT) in patients with sarcoma of the extremities treated with limb-sparing surgery is to achieve local control [23]. In the review by Roeder et al., 5-year LC rates ranged from 82 to 97% and treatments that included IOERT consistently achieved excellent limb preservation rates (81–100%) with good functional outcomes (59–100%) [54]. The ESTRO IORT Task Force recommendations, based on a literature review, were published in 2020. Those recommendations defined the indications and technical characteristics of IORT-based approaches that have been shown to provide high local control rates with low rates of acute and late toxicity [51].

### 2.4. IORT in Pancreatic Cancer

IOERT is an innovative therapeutic approach to dose intensification to improve local control in patients with locally advanced pancreatic cancer (LAPC), one of the most lethal cancer types (5-year OS < 5%) [26]. Numerous reviews have demonstrated that IOERT improves treatment outcomes in pancreatic cancer. However, because IOERT is typically administered as part of a multimodal approach, it is difficult to determine the extent to which this modality influences treatment outcomes [26,27,55]. A selection of relevant studies is reported in Table 2.

IOERT can be used in multimodal treatments as a boost strategy for dose intensification in borderline resectable disease or as the only radiation treatment for localized, non-resected, borderline, or post-resection pancreatic cancer [56,57]. IORT has been shown to improve local control in patients undergoing extended resection [58]. IOERT can be used as an individualized, risk-adapted technique to maximize locoregional control through dose escalation [59].

A non-randomized, retrospective study compared IOERT to concurrent chemoradiotherapy (CRT) in patients with LAPC [28]. Disease progression was observed in 76% of patients after IOERT and in 69.8% after CRT. No significant differences in OS were observed between the two groups (*p* = 0.458). There were fewer adverse events in the IOERT arm, with a shorter treatment time.

A systematic review and metanalysis of 15 studies (834 patients) showed that IORT significantly improved locoregional control (relative risk [RR], 0.70; 95% CI, 0.51–0.97, *p* = 0.03) and OS (median survival rate, 1.20; 95% CI, 1.06–1.37; *p* = 0.005) in patients with resectable pancreatic cancer, without increasing postoperative complications or surgery-related mortality rates [60].

The ESTRO IORT Task Force recommendations for IORT in borderline-resected pancreatic cancer and unresected pancreatic cancer were both published in 2020 [61,62]. Those articles described the treatment modalities and recommendations for IOERT in patients with close surgical margins or residual disease and in selected cases with unresectable tumors. More recently, Lee et al. evaluated immune response activity in a group of surgically treated patients with pancreatic cancer who received IORT. Patients treated with IORT showed increased levels of cytokines involved in the PI3K/SMAD pathway in the peritoneal fluid, which are associated with inhibition in the growth, migration, and invasiveness of pancreatic cancer cells [63]. The findings of that study seem to support the hypothesis that IORT activates immune mechanisms that could prevent local recurrence and, potentially, even distant relapse. Yanagi et al. recently compared patients (n = 99) diagnosed with non-metastatic pancreatic cancer who underwent one of three different treatment regimens: (1) IOERT + surgery, (2) surgery + postoperative radiotherapy (PORT), or (3) radiotherapy alone [64]. At 2-years, LC rates were 33%, 35%, and 0% in the IOERT, PORT, and radiotherapy alone groups, respectively. Five -year OS rates were 13%, 3.2%, and 0%, respectively, with no cases of grade 3–4 toxicity. Of note, distant metastases were less common in the IORT group.

Although current ESMO (European Society for Medical Oncology) guidelines recommend chemotherapy alone for LAPC, data from dose intensification studies suggest that radiotherapy should be considered as an individualized treatment option in selected patients [56]. The results of the CONKO-007 trial showed no significant differences between chemotherapy and CRT in median OS and resection rates; however, the R0 and pathologic complete response rates were both higher in the CRT group [65]. Moreover, OS was longer in the patients who underwent surgical resection. Among long-term survivors, 5-year OS rates were higher in the CRT group (especially the resected subgroup), a finding that suggests that induction chemotherapy followed by radiotherapy could potentially improve outcomes in patients with LAPC.

### 2.5. IORT in Other Cancers

In addition to the cancer types described above, IORT has also been widely used to treat several other types of cancer, usually administered as a boost in combination with pre- or post-operative radiotherapy. An analysis of the ISIORT registry described the treatment characteristics of IORT in a wide range of cancer types (gastric, esophageal, prostate, gynecological, central nervous system, skull base, head and neck, lung, kidney, and bladder) [6].

## 3. Technological Advances in Intraoperative Radiotherapy Planning and Delivery

One of the major advantages of intraoperative radiotherapy is that the radiation is delivered during surgery. Both X-rays IORT and IOERT have substantially improved over time through a series of continuous technological advances, including the advent of modern, mobile accelerators for IOERT [66,67,68]. One of the main drawbacks of IORT versus EBRT has long been the lack of high-quality images from advanced imaging techniques (CT, PET-CT, and/or MRI), which are needed to ensure accurate contouring of the volumes of interest (gross tumor volume [GTV], clinical tumor volume [CTV], and organs at risk [OAR]). High-quality images are also needed to accurately calculate the dose distribution. This drawback is especially apparent when compared to the highly advanced computerized treatment planning systems (TPS) that are now in widespread use for EBRT. These modern TPS show the isodose locations inside the body in three dimensions. This allows physicists to adjust the radiation parameters and to instantly visualize the dose distribution, which can be rapidly recalculated to suit the new patient set up [69].

During IORT, radiation oncologists and surgeons work closely together in the operating room to estimate the size and depth of the CTV based on anatomical landmarks in the surgical bed. In some cases, ultrasound probes, which permit visualization of deeper body structures, are used to ensure radiation sparing [70]. However, the accuracy of this process is highly reliant on the clinical experience of the operators, who then select the most appropriate collimator and beam energy (for electron beams). Unfortunately, in IORT, the true dose distribution in the body cannot be visualized at the time of dose application due to the lack of high-quality images, as CT and MRI scanner are not usually available in the operating room. However, the dose distribution could be easily modified, similar to EBRT, if the necessary images were available. In this regard, the introduction of hybrid operating rooms equipped with CT and MRI allows clinicians to obtain real-time images of the patient’s anatomy. While these images are essential to accurately plan IOERT, they are also useful for the surgical intervention. This allows for rapid contouring of the target and non-target structures to create a treatment plan based on real-time data. Clearly, real-time imaging is essential for optimal treatment planning. The integration of navigation systems with IOERT systems could significantly enhance the accuracy and efficiency of collimator setup.

Several reviews have shown that in-room CT imaging increases dose calculation accuracy because the dose distribution is based on a real-time assessment of the patient’s anatomy [65,66,70,71]. CT imaging is crucial because some tissues and/or structures are temporarily displaced to avoid the radiation beam. While in-room imaging is a prerequisite to improving IORT, some technical constraints associated with real-time 3D treatment planning (i.e., visualization of the dose distribution for individual patient anatomy) still need to be resolved. To determine the combined effect of IORT and EBRT doses, deformable registration systems are needed to map the dose distribution based on the patient’s anatomical status immediately prior to the second treatment [72,73]. Even after resolving the issues related to differences in terms of tissue arrangement between IORT and EBRT, the distinct biological effects per dose unit in IORT and EBRT—delivered on different time schedules—must be addressed. These considerations should be approached in a manner similar to that used for the combination of brachytherapy and EBRT.

CT scans are required to perform dose calculations in the target location, which must take into account the current clinical and anatomical status of the patient during the operation. An indirect solution is to simulate the applicator position on CT scans taken before the procedure (virtual planning). This approach may be sufficiently accurate, but only if accurate modeling algorithms (a type of AI) can be developed. At present, the most accurate, direct dose calculation method is to perform imaging immediately prior to or after tumor resection [72]. Ideally, the CT scan would be performed in the operating room. However, if in-room scans are not available, the patients could be transferred under anesthesia to another room. The drawback of this approach is that the imaging room must provide a sterile environment (similar to that of the operating room). Clearly, this approach would increase the complexity of the procedure and require more time. A better solution would be to use an in-room imaging system to perform image-guided IOERT, which could include ultrasound, cone-beam CT, or CT [74,75,76].

To accurately represent the patient’s anatomical status, images must be acquired with the collimator in place and positioned as it would be during treatment. This requires a CT scanner with a bore large enough to cover the entire treatment region—including the patient and the applicator in situ—without risking collision with the treatment table or surgical accessories. The CT scanner must also provide images of sufficient quality to allow for real-time electron beam dose calculations. To minimize imaging artifacts, the applicator should be made of non-metallic materials [77].

Target delineation is an essential aspect of IORT to ensure precision. However, contouring can prolong the surgical procedure and could negatively influence the quality of the intervention by allowing fluid to accumulate in the wound, which alters the geometry. In addition, the extra time required could affect the overall performance of the unit by reducing the number of procedures that can be performed in a given time period. This disadvantage could be overcome by using automated contouring methods. Several different methods are available, including advanced algorithms to compare differences in densities and deep learning approaches that use previously compared images as a learning set. The main obstacle to using deep learning (a type of AI) is the need for a reasonably large learning set (i.e., a large number of cases).

### Automated Segmentation and Instant Dose Distribution in IORT: Advances in AI

Computers have long been used to perform dose calculations, although there is always a degree of uncertainty. For this reason, the process might better be described as dose prediction. Even when the calculations are based on well-established formulas, small errors in the input parameters can negatively affect the results. In this regard, an important advantage of modern computers is that they are not limited to pre-set algorithms but can actually develop their own algorithms or models through “repeated options checks” to predict the development of events. The more data available and the more frequently these patterns are checked against known results (the learning set), the more accurate the results become. The accuracy is highly dependent not only on the size of the learning set and the calculation capacity of the computer, but also on the relevance of the learning set used to train the model.

Due to the major technical advancements that have been made in recent years, artificial neural networks (ANNs) can now match—and even surpass—human neural networks in terms of accuracy. While ANNs have long had the ability to process previously structured data (selected parameters), they only recently gained the capacity to process raw data, especially images, which represented an enormous advance in their processing capacity; similarly, the development of convolutional neural networks (CNNs) is considered pivotal, especially for image analysis [78,79]. Deep learning from unstructured data (raw images) is a major development, as it permits images obtained from diverse sources to be used for training (“big data” approach). This process is a clear example of artificial “intelligence”, as it is similar to how humans draw conclusions.

In the last decade, the implementation of AI applications has had a substantial impact on radiotherapy. AI can optimize treatment planning, greatly reducing the time needed to segment tumors and organs at risk and to perform treatment planning. Similarly, AI has important potential applications in quality control and assurance of treatment plans. It can also be used to assist in performing image-guided RT, which is especially useful to monitor mobile tumors during treatment. Another potential application of AI is for prognosis and prediction of tumor outcomes and side effects, with radiomics being a prominent area of research [80,81].The increased potential to closely integrate computers into decision-making processes has raised concerns about the appropriateness of learning datasets. For instance, if the images used to create the learning dataset do not adequately cover all scenarios, the resulting machine-based segmentation may not be correct. In turn, low quality training datasets that are not fitted to the real data might introduce bias and systematic errors, thereby reducing the accuracy and reliability of clinical predictions [8,82]. One solution to overcome this challenge would be to increase the number of training cases through multicenter data sharing (e.g., federated learning) [82,83]. However, in practice, only a relatively small number of centers are actively performing IORT, and an even smaller number are using intraoperative CT imaging. Moreover, inter-center differences in imaging protocols, treatment planning, and contouring practices increase the heterogeneity of the available training data, making these data less reliable.

Another challenge related to AI is that it requires advanced infrastructure (technical and computational requirements), which can limit its availability at some centers. In addition, the lack of regulations on the use AI and the current domination of “black box” models in deep learning makes it difficult to fully understand how these models work, thus undermining clinical trust [80,84]. Another obstacle is related to patient data protection (e.g., GDPR) for AI development, as models rely on large and diverse datasets require to fulfill strict regulatory compliance [79].

If the human responsible for supervising the proposed segmentation (the physicist or physician) fails to detect this error, the patient may be harmed. Currently, AI can perform some of the steps in the radiotherapy process, including segmentation (OARs and target), deformable image registration, and treatment planning. AI can also be used in image-guided and adaptive radiotherapy as well as to predict treatment response and determine prognosis, which may have an important potential impact on patient selection [73]. However, all of these applications require large, relevant learning datasets.

There are many benefits to automated segmentation. First, manual segmentation is a highly time-consuming process, and automated segmentation can reduce this from hours to mere minutes. Moreover, segmentation is highly dependent on the individual radiation oncologist (high interobserver variability) and can be affected by the number of sections, the complexity of the involved structures, and the availability of support software. One study found that AI-assisted EBRT reduces segmentation time by 65% (5.4 min) and decreases inter-observer variability for the GTV by 32% [85]. These findings are relevant in the surgical setting in which IORT is performed due to the importance of limiting the time required for target delineation following image acquisition. In EBRT, delineation is performed prior to treatment and can be performed manually because time constraints are less important than in IORT. Even so, AI-assisted software is increasingly employed in EBRT.

The development of CNNs has advanced segmentation beyond traditional approaches (intensity analysis, shape modeling, and atlas-based methods) by enabling the automatic extraction of hierarchical features directly from imaging data. The U-Net architecture has emerged as the most widely applied model in radiotherapy segmentation, characterized by its contracting and expanding paths—an encoder and a decoder—that together form the distinctive U-shaped structure [79,86]. However, several commercial solutions also use CNNs for segmentation, most notably MVision (Helsinki, Finland), AutoContour (Radformation, New York, NY, USA), RayStation (RaySearch, Stockholm, Sweden), and Annotate (Therapanacea, Paris, France) [83].

In IOERT, contouring must be performed during surgery, with an open wound susceptible to infection. In this setting, time is a crucial factor.

In IORT, the target volume is the surgical bed, with the surrounding healthy tissues temporarily displaced to permit direct irradiation of the target. As a result, the tissue anatomy depends on the time point (i.e., preoperative, intra-operative, and after suturing) [75]. Thus, there is a clear need for automated support to reduce the time needed for contouring, ideally to only a few minutes.

In EBRT, automated segmentation is based on large databases of image atlases. Similar imaging databases are needed for IORT, especially because the shape and volume of the body structures can be substantially altered during surgery. This loss of body integrity alters the relationships between organs and tissues and must be accounted for during IORT.

Deep learning methods, based on raw images acquired pre- and post-resection, may enable AI to be used to automatically segment key structures and to rapidly perform the dose calculation. In this approach, images obtained preoperatively are combined with images acquired immediately after surgery (assuming the operating room has a CT scanner). Over time, we may eventually have a sufficiently large learning set. At present, however, it is not clear exactly how these self-developed patterns—in which the learning set is based on numerous preoperative cases but many fewer postoperative cases—will work. Moreover, the accuracy of these patterns is also uncertain. In most cases, in-room CT and MRI-based imaging is not available, and thus the radiation parameters for IORT are selected without the assistance of these images or isodoses. Under these conditions, AI-based automated segmentation seems to be justified. Moreover, since automated segmentation can be completed in only a few seconds and the dose distribution calculated nearly instantly, the applicator can be placed in the optimal location to avoid irradiating healthy tissues.

Although anatomic variability represents a major challenge in using AI for segmentation and treatment planning during IORT, the wide variety of surgical procedures in use is another important obstacle.

The validity of learning datasets in large population settings is an unresolved question because a dataset acquired from one population may not be relevant to a different population due to differences in body build, including height, weight, and other anatomical differences (body mass index, obesity, etc.). In this sense, the dataset should take heterogeneity in these parameters into account, many of which are related to differences in age, sex, and body structure. In the future, it may be possible to use AI to create, prior to treatment, virtual reality-based representations of the patient’s probable anatomical status during IORT. This representation could include expected alterations to the organs and any displacements related to open surgery or minimally invasive laparoscopic or robotic procedures.

AI can have a large impact on dose distributions in IOERT. Knowledge-based treatment planning (KBP), a machine learning model, was first introduced in 2014 and is now widely used in IMRT [76]. In this model, data from prior treatment plans are registered and then used to create a semiautomatic plan, thus significantly reducing the time needed for treatment planning. Commercial systems such as RapidPlan from Varian and RayStation from RaySearch apply KBP to create consistent, high-quality automated treatment plans [87,88]. More recent developments include deep learning methods capable of generating new dose distributions based on previous treatments plans, offering the potential to rapidly deliver and optimize treatment planning, in some cases in a question of minutes or even seconds [77,78,87,89]. In DL-based approaches (such as U-Net models), dose estimation is possible to directly predict dose distributions and/or monitor units, leaf positions, and jaw positions for VMAT plans, eliminating the need for traditional planning methods [88,90]. “Intraplanning”, a technique in which the treatment plan is adapted to the patient’s real-time anatomy, may become the standard approach to planning in the next generation of IOERT TPS [91].

AI can also support the management of internal body movements that impact target positioning during radiotherapy. For example, in breast cancer treatments, AI-driven systems can account for the respiratory phase in breath-hold settings or help reduce respiratory motion, thereby minimizing or preventing target displacement during irradiation. Similar strategies can be applied to thoracic and upper abdominal cancers. A specific aspect of IORT is related to artificial ventilation during general anesthesia, which makes respiratory movements more reproducible than during spontaneous respiration, therefore more open to modeling. Irradiation of the lower abdomen typically involves relatively high dose rates, which means treatment is short and bowel movement-related anatomical changes are limited. Similarly, bladder filling is typically managed by catheterization. For cancers located in the head and neck region or the extremities, no significant changes due to internal body movements are expected during IOERT.

AI is currently used in EBRT to help determine prognosis through radiomic analysis of imaging studies. AI could be used in the same way for IORT. It could also be used to help identify the patients most likely to benefit from IORT. Individual radiation sensitivity is also relevant to IORT. Several different molecular methods are used to evaluate resected tissues (including cells from the tumor microenvironment). It is now possible to analyze molecular data from both cancerous and non-cancerous cells, enabling the use of these datasets to train machine learning and deep learning algorithms. Furthermore, these molecular data can be integrated with information on dose distribution and side effects to help personalize IORT. Although researchers are actively developing such predictive models, no validated models are currently available [92,93].

Machine support and automation have long been used in industry—particularly in aviation—and now play a well-established role in radiotherapy, where they can greatly reduce the time needed for clinical decision-making. These tools help medical physicists and physicians to optimize treatment-related decisions and to adapt the treatment to suit the individual patient’s needs. However, as automation takes on a greater role in the decision-making process, there is a growing risk of liability. At present, clinical decisions are evaluated according to the available information and on adherence to well-established good practices. If a clinical decision based on a machine-generated pattern turns out to be incorrect, there may be no protocol to address this type of error. In current clinical practice, machine-generated contouring must be reviewed and approved by a human, thus ensuring adherence to clinical protocols and good clinical practice. However, in the IORT setting, this practice is more challenging due to the real-time nature of this technique, in which there is only a short window of time to assess AI-generated recommendations. In turn, these advances will allow for better clinical decision-making based on the unique properties of the specific tumor and individual patient. These datasets could potentially also be reused to help in developing treatment plans for external radiotherapy, if necessary.

One of the main limitations of this study is the appropriateness of the available learning datasets in terms of size and case characteristics. These datasets are based on highly heterogeneous patient populations, which may affect the validity of a specific learning dataset due to differences in age, sex, and body structure. Moreover, it is important to account for the impact of different surgical approaches on patient anatomy. In any case, it is clear that all of the steps performed by AI tools must be carefully reviewed and approved by the professionals involved in the clinical and technical aspects of the radiotherapy process, including contouring and dose delivery.

## 4. Conclusions

Intraoperative radiotherapy plays an important role in radiotherapy, offering numerous well-documented advantages in several types of cancers. Given the current capabilities of modern computer systems, together with recent advances in machine-generated decision-making patterns, it is clear that AI could greatly improve various aspects of IORT (as is currently performed in EBRT), most notably real-time segmentation and isodose calculations.

In the near future, AI may be used to facilitate patient selection and to predict clinical response and radiation-induced complications in IORT by taking into account individual risk factors such as genetics, environmental factors, and lifestyle habits. This approach to the wider application of AI could help to expand the use of intraoperative radiotherapy, which is a highly remains a promising cancer treatment modality that could be combined with anticancer agents such as immunotherapy to further improve treatment outcomes.

## Figures and Tables

**Table 1:** Differences between intraoperative irradiation techniques: low-energy X-ray versus electrons.

	IORT by Low-Energy X-Rays (Spherical Applicator)	IORT by Electrons (6–12 MeV)
Dose at surface (% of maximum dose)	Higher (100%)	Lower (86–94%)
Dose at 2 cm depth	Lower (<20%)	Higher (70–100%)
Dose homogeneity (surface to depth)	>150% variation	<15% variation
Depth of useful dose	0 cm–1 cm	Up to 4–5 cm
Treatment time (or Beam-on time)	30–45 min	2–4 min
Procedure time	45–120 min	30–45 min
Treatment sites	Small target volume within 1 cm from the applicator surface	Larger target volume accessible with applicators of about 10 cm in max diameter

**Table 2:** Selected clinical trials of IORT and IOERT for breast cancer, rectal cancer, sarcoma, and pancreatic cancer.

Tumor Site	Author/Year	Series	Study Design	Modality	IORT Dose (Gy)	Sample Size	Key Outcomes
Breast	Fastner et al., 2013 [15]	ISIORT, Multi-institutional	Observational (pooled analysis)	IOERT boost	10	1109	LC 99.2% after median F/U of 72.4 months
Breast	Orecchia et al., 2021 [16]	ELIOT, Mono-Institutional	Phase III	IOERT vs. WBRT	21	1305	15-yr LR: WBRT 2.4% vs. 10.2% IORT %. *p* = <0.001; in lower-risk pts no significant difference for LR
Breast	Vaidya et al., 2023 [17]	TARGIT-A, Multi-institutional	Phase III	Intrabeam IORT vs. WBRT	20	2298	No statistically significant difference for LC
Rectum	Calvo et al., 2002 [18]	Mono-institutional, LARC	Observational	CRT + surgery + IORT	12	100	1 in-field IORT failure; 14 distant failures
Rectum	Kusters et al., 2010 [19]	Multi-institutional, LARC	Observational (pooled analysis)	CRT + IORT + CT	12.5	605	LR rate 12.5%
Rectum	Masaki et al. 2010 [20]	Mono-institutional	Phase III	Preserved bilateral pelvic plexus + IORT vs. partial bilateral pelvic plexus preservation without IORT	18–20	76	Higher number of distant metastases in IORT patients (*p* = 0.04)
Rectum	Dubois et al., 2011 [21]	Multi-institutional, LARC	Phase III	Pre-RT + surgery ± IORT	18	142	No benefit for IORT in local control or survival
Sarcoma	Sindelar et al., 1993 [22]	NIH, Mono-institutional, RPS	Phase III	IORT + Low dose EBRT versus High dose EBRT	20	35	IORT patients experienced lower toxicity; LC 6/15 IORT; 16/20 EBRT
Sarcoma	Calvo et al., 2014 [23]	Multi-institutional, Extremities, limb sparing	Observational (pooled analysis)	IORT + EBRT	10–20	159	5-year IOERT in-field control 86%, DFS 61%, OS 72%
Sarcoma	Roeder et al., 2018 [24]	Multi-institutional, RPS	Observational	IORT+/− EBRT	15	156	5-year OS 63% in the primary situation and 68% after R0 resection
Sarcoma	Seidensaal et al., 2023 [25]	Mono-institutional, RPS	Phase I/II	IMRT + IORT boost	12–20	37	Primary endpoint (5-year LC = 70%) was not met
Pancreas	Tepper et al., 1991 [26]	RTOG, Multi-institutional series	Observational	IORT + EBRT + chemo	20	51	Median OS 9 months; LC not assessed
Pancreas	Valentini et al. 2009 [27]	ISIORT, Multi-institutional	Observational (pooled analysis)	Surgery + IORT +/− EBRT	15	270	5-yr LC 23.3%
Pancreas	Ren et al., 2021 [28]	Multi-institutional, LAPC	Retrospective, CCRT vs. IORT	15–20 Gy	15–20	103	G3-G4 toxicity higher in CCRT pts (34% vs. 0%)

Abbreviations: IOERT: Intraoperative electron radiotherapy; WBRT: Whole breast irradiation; LR: Local recurrence; LC: Local control; R0: Complete excision; EBRT: External beam radiotherapy; RPS: Retroperitoneal sarcomas; IMRT: Intensity modulated radiotherapy; DFS, Disease-free survival.

## Data Availability

No new data were created or analyzed in this study.

## Abbreviations

The following abbreviations are used in this manuscript:

IORT	Intraoperative radiotherapy
AI	Artificial Intelligence
IOERT	Intraoperative electron radiotherapy
ISIORT	International Society of Intraoperative Radiation Therapy
CT	Computer tomography
LC	Local recurrence
IBTR	In-breast tumor recurrence
APBI	Accelerate partial breast irradiation
WBI	Whole breast irradiation
PBI	Partial breast irradiation
LARC	Locally advanced rectal cancer
LAPC	Locally advanced pancreatic cancer
GTV	Gross tumor volume
CTV	Clinical target volume
EBRT	External beam radiotherapy
TPS	Treatment planning system
MRI	Magnetic resonance
ANN	Artificial neural networks
OAR	Organ at risk
IMRT	Intensity modulated radiotherapy
